# A Phase I Randomized Trial of Topical Insulin for Glaucoma: Safety and Efficacy Outcomes

**DOI:** 10.1016/j.xops.2025.101032

**Published:** 2025-12-06

**Authors:** Mariella Saludares, Zachary Wennberg-Smith, Gala Beykin, Tasneem Z. Khatib, Mariana Nunez, QianQian Wang, Adriana Di Polo, Jeffrey L. Goldberg

**Affiliations:** 1 Spencer Center For Vision Research, Byers Eye Institute at Stanford, Department of Ophthalmology, Stanford University School of Medicine, Stanford, California; 2 Universite de Montreal, Montreal, Quebec, Canada

**Keywords:** Glaucoma, Glaucomatous optic neuropathy, Neuroprotection, Optic nerve, Retinal nerve fiber layer

## Abstract

**Purpose:**

Insulin is neuroprotective in mouse and nonhuman primate models of glaucoma. Here we evaluate the safety and efficacy of topical insulin in human patients with glaucoma.

**Methods:**

This prospective study of once-daily topical insulin for glaucoma was conducted in 2 parts: an open-label dose escalation phase evaluating low (100 units/mL) and high doses (500 units/mL), followed by a randomized masked trial comparing low- and high-dose topical insulin. The worse-affected eye served as the study eye while the contralateral eye served as a control. The primary endpoint was safety and tolerability, monitored by recording adverse events (AEs), intraocular pressure, and serum blood glucose and potassium levels. The secondary endpoints included structural and functional testing including visual acuity, 24-2 Humphrey visual field (HVF), OCT, and macular and peripapillary flavoprotein fluorescence (FPF).

**Results:**

Safety and tolerability were favorable for topical insulin at both doses, with no serious AEs. The most frequent AE was transient stinging, resolving almost immediately after application to the study eyes. Blood glucose levels remained consistently stable before and after insulin administration for all groups. In group 3, 5 of 15 study eyes showed an increase of average retinal nerve fiber layer (RNFL) thickness by ≥5 μm, compared with 0 of 12 fellow eyes from baseline to the 1-month visit (*P* = 0.0476). On average, study eyes showed a larger increase in RNFL thickness compared with fellow eyes (1.11 μm in fellow eyes vs. 2.50 μm in study eyes) with no evidence of cystoid edema, and no difference detected between the 2 dose levels. The RNFL increase reversed to baseline by 1 month after cessation of insulin treatment. There were no changes in HVF or FPF during a 4-week administration period.

**Conclusions:**

The application of topical insulin proved safe and well-tolerated among patients with glaucoma. Notably, 1 month of topical insulin led to a significant, reversible increase in RNFL thickness, indicating evidence for pharmacodynamic effect at the retina after topical delivery. These data support study initiation to evaluate topical insulin's neuroprotective or neuroenhancement effects.

**Financial Disclosure(s):**

Proprietary or commercial disclosure may be found in the Footnotes and Disclosures at the end of this article.

Glaucoma is the leading cause of irreversible blindness worldwide, affecting an estimated number of 64.3 million people worldwide in 2013, which is predicted to rise to 111.8 million in 2040.^1^ This neurodegenerative disease is characterized by damage to retinal ganglion cells (RGCs), the output neurons of the retina, which can result in significant vision loss.^2^ Current glaucoma treatments typically include medical and/or surgical interventions that lower the intraocular pressure (IOP).^3^ Despite efforts to reduce IOP, a significant number of patients still endure vision loss.^4^ Therefore, the exploration of IOP independent neuroprotective and/or neuroregenerative therapies that address glaucoma's pathophysiology, specifically aiming to preserve RGCs, is crucial.

Insulin is a peptide hormone that has a well-established role in maintaining glucose control.^5^ In addition to this primary role in metabolic homeostasis, insulin can bind to insulin-like growth factor receptors that are found throughout the human body, including the retina, which are involved in a signaling cascade that can promote neuronal cell survival and other functions.^6^^,^^7^ Even in the absence of diabetes, insufficient insulin signaling has been linked to neurodegenerative diseases such as Alzheimer and Parkinson diseases, and notably, glaucoma.^8^ Preclinical studies in a mouse model of glaucoma have shown that human recombinant insulin delivered topically or systemically prevents RGC apoptosis and promotes neuroregeneration.^9^^,^^10^ These preclinical data suggest human recombinant insulin may serve as a promising treatment for optic neuropathies such as glaucoma.

No previous trials have tested the safety and efficacy of topical human recombinant insulin in human subjects with optic neuropathies. In this study, our primary focus is to evaluate the safety of topical insulin use in patients with glaucoma and examine the impact of insulin on both structural and functional measures.

## Methods

### Study Design

This is a phase I, dose-escalation, safety study of topical sterile human insulin in patients with glaucomatous optic neuropathy. This single-center, prospective study, conducted at the Byers Eye Institute at Stanford University, registered at ClinicalTrials.gov (ClinicalTrials.gov identifier: NCT05206877), was reviewed and approved by the appropriate institutional review board, and conducted in accordance with the principles of the Declaration of Helsinki, and in compliance with US Food and Drug Administration Good Clinical Practice guidelines. After informed consent was obtained, participants with the diagnosis of glaucoma were sequentially enrolled from April 2022 to August 2022. One patient with nonarteritic anterior ischemic neuropathy was treated with this protocol but is not reported here. Patients with glucose intolerance or those diagnosed with type I or type II diabetes mellitus were excluded from the study due to the unknown of how additional insulin exposure could affect such patients in this is phase I study. Other specific inclusion and exclusion criteria are outlined in Table 1. For each participant, the eye with the worse glaucomatous damage by visual acuity and/or Humphrey visual field (HVF) was selected as the study eye for safety and ethical reasons consistent with a phase I study. There was no exclusion regarding prior intraocular surgery for the study eyes. Participants were kept on their normal IOP-lowering medications throughout the study. The study did not prohibit any standard of care changes to IOP lowering during enrollment. The patient's fellow eye was used as a control in the study. Five participants were enrolled in group 1 and received 100 units/mL of sterile human recombinant insulin (Humulin R U-100, Eli Lilly), 4 units per drop, 1 drop delivered in clinic daily for 5 consecutive days. After group 1 was completed and analyzed for safety, 6 participants were enrolled in group 2 and received a higher dose of 500 units/mL (Humulin R U-500, Eli Lilly), 20 units per drop, 1 drop delivered in clinic daily for 5 consecutive days. Three participants from group 1 re-enrolled in group 2. There was no concern for carry-over effects due to the length of time that occurred between participation in each of the groups.

**Table 1:** Study Inclusion and Exclusion Criteria

Inclusion criteria	• Capable of providing informed consent • Age ≥18 yrs • Diagnosed with optic neuropathy, either glaucoma, nonarteritic ischemic optic neuropathy, or optic disc drusen • Only 1 eye per patient will be selected as the study eye—if both eyes meet inclusion criteria, the eye with the worse acuity and/or visual field was selected.
Exclusion criteria	• Pregnancy or breastfeeding • Presence of other ocular pathologies that contributes to severe vision loss (retinopathy/maculopathy, severe uveitis, keratopathy, etc.) • Diagnosis of glucose intolerance, type 1 or 2 diabetes mellitus (diagnosis of “prediabetic” allowed) • Inability to perform a reliable visual field • Unable to complete the tests and follow-ups required by the study

After safety analysis of groups 1 and 2, a randomized, masked group 3 screened 22 patients and enrolled 19 (3 screen failures: 2 patients did not meet inclusion criteria, and 1 patient decided to not participate). Five patients from groups 1 and 2 were re-enrolled in group 3. Two patients withdrew during the study due to worsening dry-eye symptoms, leaving 17 patients that successfully completed the study (89% study participant completion). Participants were randomly assigned (1:1) to receive either the low-dose insulin (100 u/mL) or high-dose insulin (500 u/mL), self-administered for 28 days. Eight patients were assigned to low-dose and 9 patients were assigned to high-dose. In all 3 groups, participants were permitted to maintain their normal diets throughout the trial.

### Study Activities and Assessments

The primary endpoint of safety was closely monitored by measuring adverse events (AEs), IOP, blood glucose levels, hemoglobin A1c (HbA1c) levels, and potassium levels for all patients before, during, and after treatment. Intraocular pressure was measured using Goldmann applanation tonometry before topical insulin administration for groups 1 and 3. To evaluate whether the insulin caused any potential symptoms in group 2 participants, we measured IOP using the iCare device (Tiolat Oy), which does not require the use of a topical anesthetic, unlike traditional tonometry methods, and therefore is better suited to identify ocular surface symptoms.

For groups 1 and 2, on each of the 5 days of treatment, glucose levels were measured using a handheld glucometer (TD-4116; Metene) immediately prior and 2 hours postinsulin administration. All other testing and measurements occurred at baseline and day 5 of insulin treatment (i.e., serum electrolyte and HbA1c levels). Dietary intake was not controlled or monitored, enabling the assessment of glucose levels throughout the day as experienced without additional instruction. For randomized group 3, participants underwent all testing and measurements at baseline, end-of-treatment month 1, and month 2.

The secondary endpoints of efficacy were measured by Snellen visual acuity, anterior and posterior segment slit lamp examination, 24-2 and 10-2 HVF using the Swedish Interactive Threshold Algorithm–Standard, and OCT (Cirrus; Carl Zeiss Meditec) of retinal nerve fiber layer (RNFL) and ganglion cell complex (GCC), and macular and peripapillary flavoprotein fluorescence (FPF) scores obtained by a filtered fundus camera (OcuMet Beacon, OcuSciences Inc).

### HVF

To measure visual field sensitivity, the 24-2 Swedish Interactive Threshold Algorithm Fast algorithm was used for both eyes at each study visit; 10-2 testing was conducted for patients with advanced disease, defined as a mean deviation (MD) worse than –12 decibels (dB) or any points in the central 5° on the global plot equaling 0 dB.^11^

For groups 1 and 2, all HVF data were included in analysis, regardless of false-negative or false-positive percentages, if data were available for both baseline and day 5 visits.

In group 3, 12 of 17 patients' study eyes (71%) were included in analysis after successfully completing reliable HVF exams at all 3 visits. The fellow eye of 9 of 17 patients (53%) were also included in the study analysis. Humphrey visual field data were excluded from analysis if any of the following criteria were met: (1) false-positives >20%; (2) false-negatives >35% for an MD worse than –12 dB; and (3) false-negatives >25% for an MD better than –12 dB.^12^ Additionally, an eye was excluded from analysis if reliable data were not available for all 3 visits (baseline, month 1, or month 2).

### OCT

Retinal structure was assessed by evaluating changes in RNFL and macular thickness which was also segmented to analyze GCC by OCT (Cirrus, Zeiss Meditec). The scans were analyzed using the built in OCT review software and data was exported via Zeiss Forum viewer (Zeiss Meditec). OCT scans were performed by certified technicians.

In group 3, 15 of 17 patients' study eyes (88%) and 12 of 17 fellow eyes (71%) were included in the RNFL data analysis. If an RNFL scan at any of the 3 time points resulted in a weak signal strength <6, data loss, or segmentation error, the patient's data were excluded from analysis (2 study eyes and 5 fellow eyes were excluded).

### OcuMet

A metabolically targeted imaging device was employed to explore the potential impact of oxidative stress and mitochondrial dysfunction, which play a potential role in the pathophysiology of retinal diseases, including glaucoma.^13^ When oxidative stress occurs in the eye, the flavoproteins in mitochondria will emit green light when excited by blue light.^13^ By using a filtered fundus camera, FPF scores were obtained, indicating the amount of green light emitted by susceptible mitochondria. Studies show that higher FPF scores correlate with disease severity, as determined by visual field tests and RNFL thickness measurements.^14^

### Statistics

Subgroup and overall analyses were performed for all 3 groups. Standard statistical methods were used to evaluate outcome variables with these merged data sets. GraphPad Prism software was used for statistical analysis. All relevant data are shown using the means and the standard error calculated in Excel (Microsoft). Group-level statistics on RNFL thickness were calculated using Fisher exact test. Correlation of macular thickness and RNFL was calculated on Excel using a standard linear trendline with reported R^2^ value. We provided descriptive statistics as specified in the text and included patient-level data where relevant.

## Results

### Safety Outcomes

**Figure 1:**
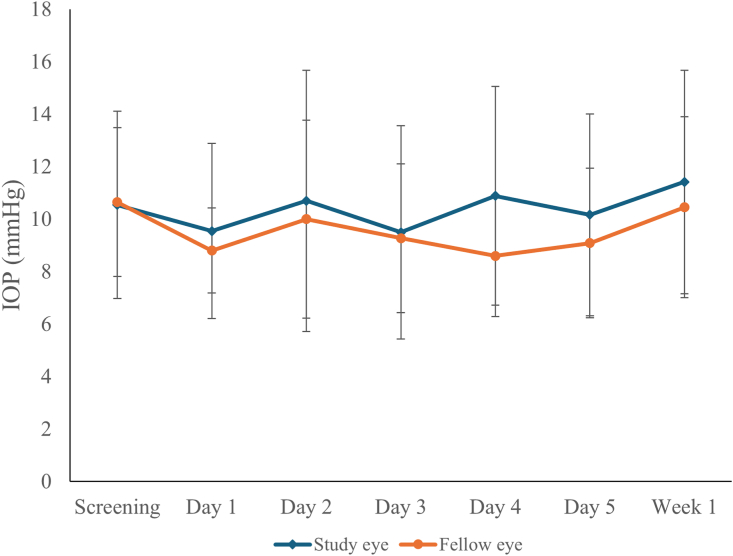
Intraocular pressure measured on study and fellow eyes over the course of groups 1-2 participation. Mean ± standard deviation shown; N = 12 eyes per group at all time points. IOP = intraocular pressure.

A total of 21 unique patients were enrolled across all 3 groups. Baseline study population characteristics are tabulated in Table 2. Topical insulin drops dosed daily at either concentration were tolerated well. No serious ophthalmic AEs due to the topical insulin drops were recorded. Most of the AEs were mild and included stinging, watery eyes, and dry-eye sensation, all of which subsided almost immediately after application (Table 3). One patient with chronic dry eye reported worse symptoms and withdrew from the study. Intraocular pressure was stable for both study and fellow eyes throughout the study duration (groups 1-2 shown in Figure 1). All enrolled participants were on ≥1 IOP-lowering medication. A list of the number of IOP-lowering medications and the class of medications are shown in Tables 4 and 5; no patients underwent any changes to concomitant meds during their enrollment in the study. One patient in group 3 had higher IOP thought to be unrelated to the study treatment and withdrew to pursue surgical treatment.

**Table 2:** Baseline Patient Characteristics

Characteristic	Group 1: Low Dose (N = 5)	Group 2: High Dose (N = 6)	Group 3: Randomized (N = 17)	All Patients (N = 21)
Demographics				
Sex				
Male	3 (60%)	3 (50%)	9 (53%)	10 (48%)
Female	2 (40%)	3 (50%)	8 (47%)	11 (52%)
Race				
White	3 (60%)	5 (83%)	10 (59%)	14 (67%)
Asian	2 (40%)	1 (17%)	7 (41%)	7 (33%)
Age				
Mean	63.20	67.33	63.24	64.90
Median	61	66	64	66
Range	52–86	55–86	39–76	39–86
Clinical characteristics				
Diagnosis				
POAG	2	4	11	14
Normal-tension glaucoma	2	0	4	4
Pigmentary glaucoma	0	0	1	1
CACG	1	1	1	1
Mixed mechanism glaucoma	0	1	0	1
Severity				
MD > –12 dB (mild to moderate)	0	1	10	13
MD < –12 dB (severe)	5	5	7	8

CACG = chronic angle-closure glaucoma; dB = decibels; MD = mean deviation; POAG = primary open-angle glaucoma.

“All Patients” column accounts for patients participating in multiple groups, counted only once.

**Table 3:** Number of Adverse Events for Study, Fellow, and OU

Adverse Event	Study Eye	Fellow Eye	OU	Total
Irritation/stinging	19	0	0	19
Dry-eye sensation	4	0	0	4
Watery eye	1	0	0	1
Blurred/worsened vision	2	0	0	2
Photophobia	1	0	0	1
Elevated intraocular pressure	0	0	1	1
Foreign body sensation	1	0	0	1

OU = both eyes.

**Table 4:** Number and Percent of Participants Taking Single or Multiple IOP-Lowering Medications at Baseline

Number of medications	1 Medication	2 Medications	3 Medications	4 Medications
Number of patients (%)	4 (21%)	5 (26%)	7 (37%)	3 (16%)

IOP = intraocular pressure.

Combination medicines are considered 2 different medications.

**Table 5:** Number and Percent of Patients That are Taking Each Class of Medication at Baseline

Class of medication	Prostaglandin Analog	Carbonic Anhydrase Inhibitor	Beta-Blocker	Rho Kinase Inhibitor	Alpha Agonist
Number of patients	15 (79%)	13 (68%)	12 (63%)	3 (16%)	9 (47%)

No changes in concomitant medications were noted throughout the course of the study.

**Figure 2:**
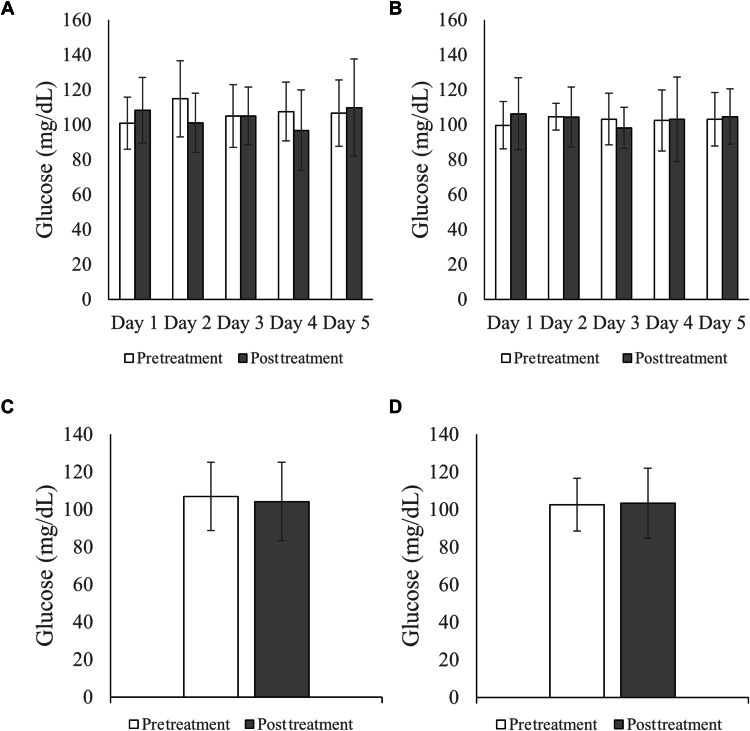
Glycemia measurements pre-insulin and postinsulin treatment for groups 1 and 2 on days 1 through 5 (panels **A-B**) or averaged across the entire week (panels **C-D**). Panels B and D exclude prediabetic outliers. Mean ± standard deviation shown; N = 11, 8, 11 and 8 in panels **A–D**, respectively.

**Figure 3:**
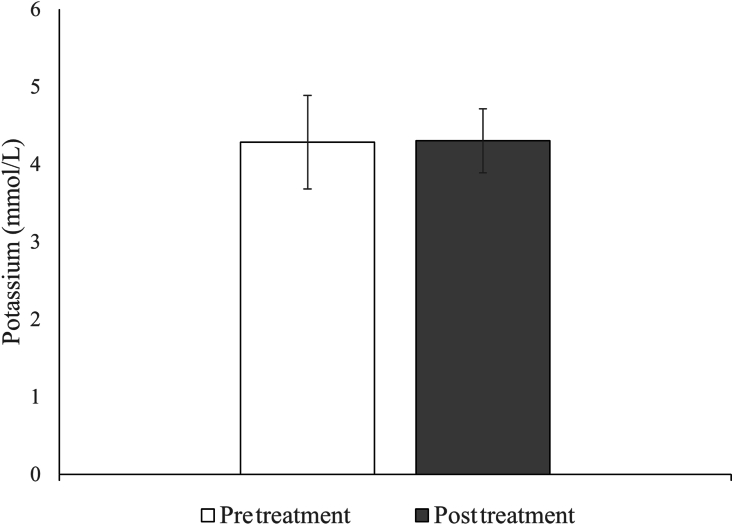
Potassium levels before and after insulin treatment for participants in groups 1-3. Mean ± standard deviation shown; N = 31.

No systemic side effects were experienced due to the topical insulin. Hemoglobin A1c levels showed no changes throughout the course of treatment for all participants. Across groups 1 and 2 participants, average blood glucose levels remained stable before (106.98 ± 18.19 mg/dL) and 2 hours after (104.21 ± 20.87 mg/dL) receiving daily topical insulin on days 1 through 5 (Fig 2). One participant with a prediabetic HbA1c value of 6.1% demonstrated a greater decrease in blood glucose levels after topical insulin delivery (pretreatment, 121.7 ± 16.73 mg/dL; 2 hours posttreatment, 100.9 ± 12.42 mg/dL). Another patient with an HbA1c value of 5.6% experienced notable fluctuations in glucose level readings on different days before and after insulin treatment. Data excluding these patients are depicted in Figure 2B, D. No patients exhibited symptoms or measurement of hypoglycemia during the trial. Average serum potassium levels across participants in all 3 groups also remained stable throughout the study duration (Fig 3).

### Functional and Metabolic Measures

**Figure 4:**
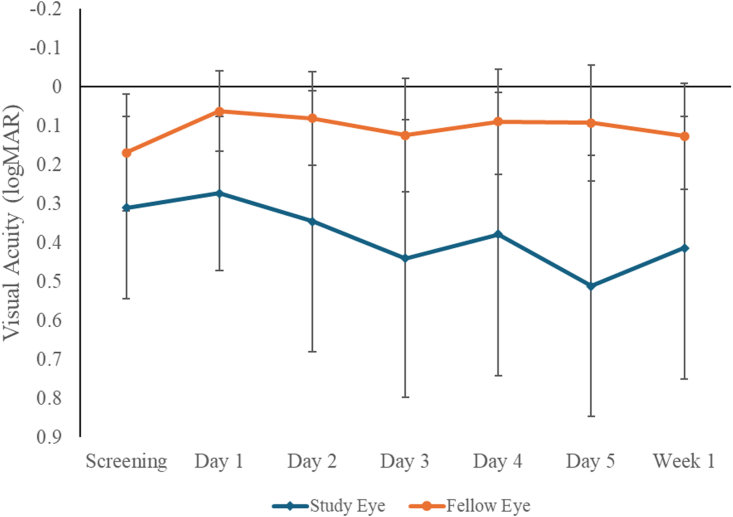
Visual acuity measured by Snellen testing converted to logMAR values on study and fellow eyes over the course of groups 1-2 participation. Mean ± standard deviation shown; N = 12 eyes per group at all time points. logMAR = logarithm of the minimum angle of resolution.

**Figure 5:**
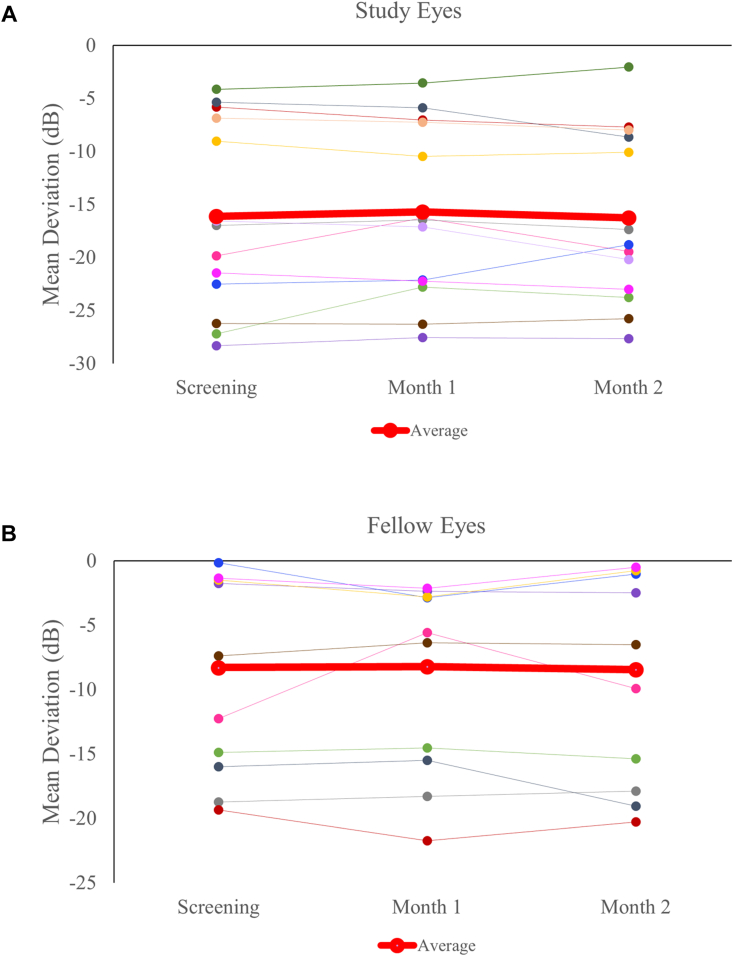
Changes in Humphrey visual field mean deviation for group 3 from baseline to month 2 for study eyes (panel **A**, N = 12) and fellow eyes (panel **B**, N = 9). Red line shows mean at each time point. dB = decibels.

**Figure 6:**
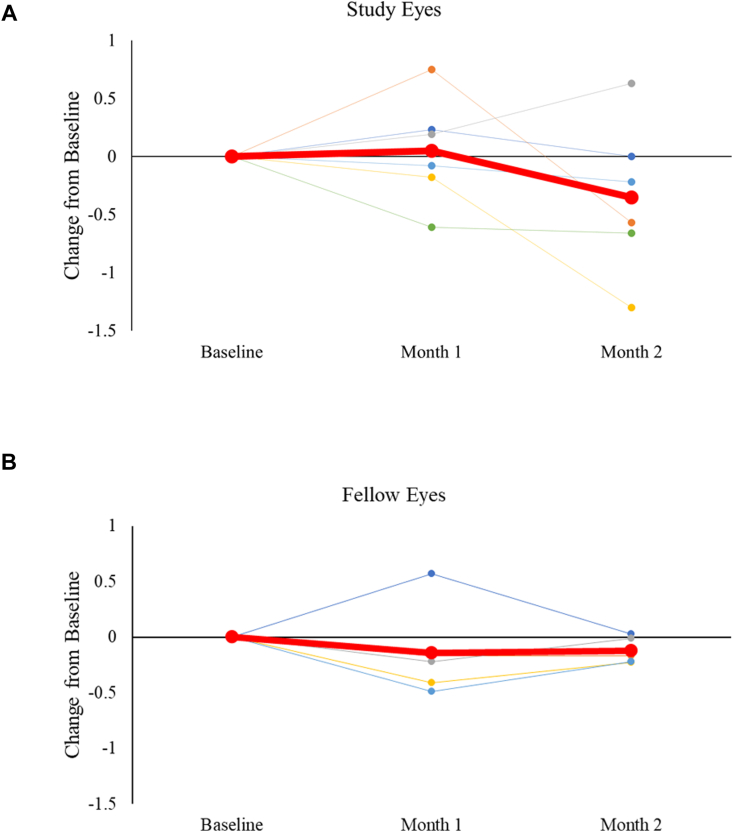
Global optic nerve head flavoprotein flourescence (flavoprotein fluorescence) change for group 3 from baseline through month 2 for study (panel **A**, N = 6) and fellow eyes (panel **B**, N = 5). Red line shows mean at each time point.

Visual acuity remained stable for both study and fellow eyes with no significant changes throughout the course of the study. Snellen scores for groups 1-2 were converted to logarithm of the minimum angle of resolution values and are depicted in Figure 4; 24-2 visual field MD was stable for group 1 and 2 participants in study eyes (pretreatment, –22.02 ± 9.53 dB; posttreatment, –20.37 ± 8.03 dB) and fellow eyes (pretreatment, –13.10 ± 11.48 dB; posttreatment, –11.91 ± 10.43 dB). Group 3 participants also exhibited stable MDs for 24-2 HVF testing, shown in Figure 5. In the data for treated and fellow eyes (n = 6 and 5, respectively, due to difficulty in obtaining reliable images caused by image artifacts such as shadowing or cataract), Ocumet FPF analysis did not demonstrate any significant changes between study (pretreatment, 5.99 ± 0.66; 1 month posttreatment, 6.04 ± 0.76, a 0.83% increase) or fellow eyes (pretreatment, 5.53 ± 0.40; 1 month posttreatment, 5.39 ± 0.52, a 2.6% decrease) as shown in Figure 6; it is not known if these changes are meaningful.

### Structural Measures

**Figure 7:**
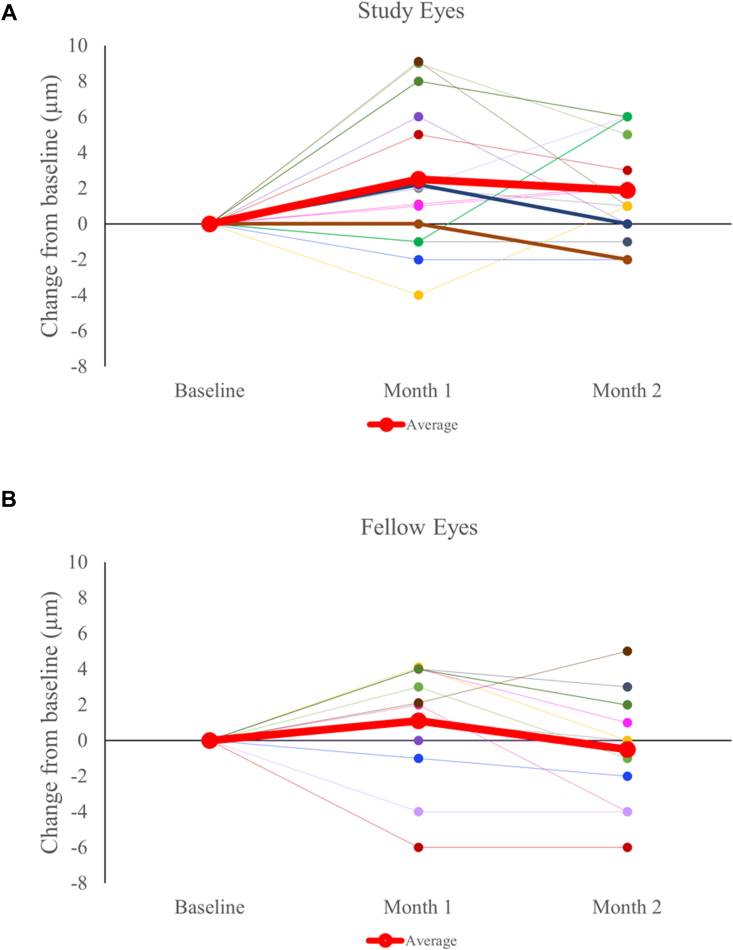
Retinal nerve fiber layer thickness change for group 3 from baseline through month 2 for study (panel **A**, N = 15) and fellow eyes (panel **B**, N = 12). Red line shows mean at each time point.

For group 1 and 2 participants, average RNFL thickness measured by OCT remained stable throughout the 5-day treatment period for study and fellow eyes. In group 3, 5 of 15 study eyes demonstrated an increase of average RNFL thickness by ≥5 μm, compared with 0 of 12 fellow eyes from baseline to the 1-month visit (*P* = 0.0476, Fisher exact test). From baseline to month 1, on average, study eyes exhibited a larger increase in RNFL thickness compared with fellow eyes, with no evidence of cystoid edema (Fig 7; fellow eyes: 1.11 μm, study eyes: 2.50 μm). This RNFL thickening at the month 1 (end-of-treatment) time point then decreased back to baseline after a washout period of 1 month (2-month time point). Subgroup analysis did not detect any substantial differences between the 100 u/mL or 500 u/mL dosages.

**Figure 8:**
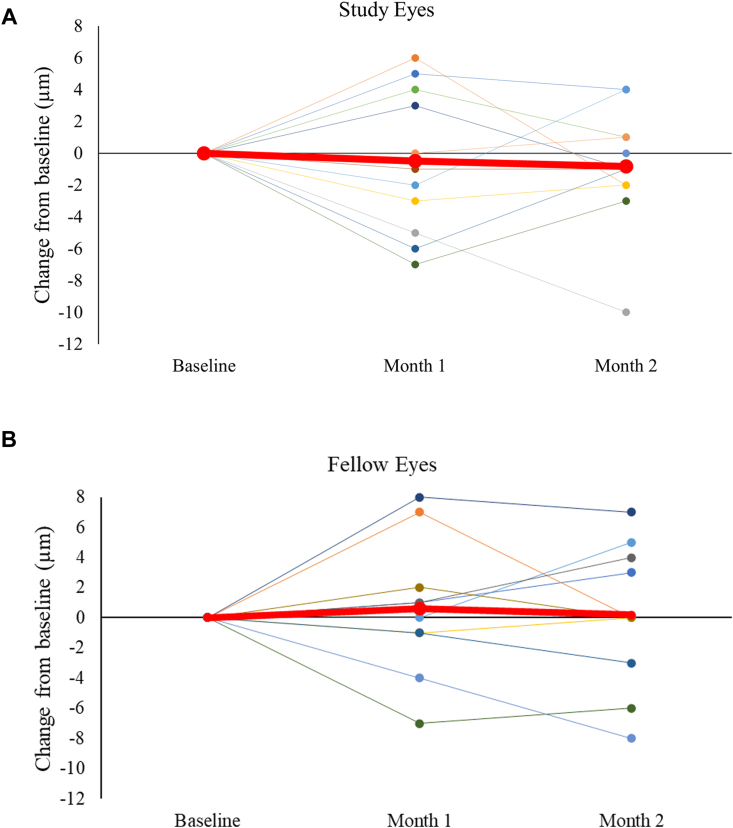
Macular thickness change for group 3 from baseline through month 2 for study (panel **A**, N = 12) and fellow eyes (panel **B**, N = 10). Red line shows mean at each time point.

**Figure 9:**
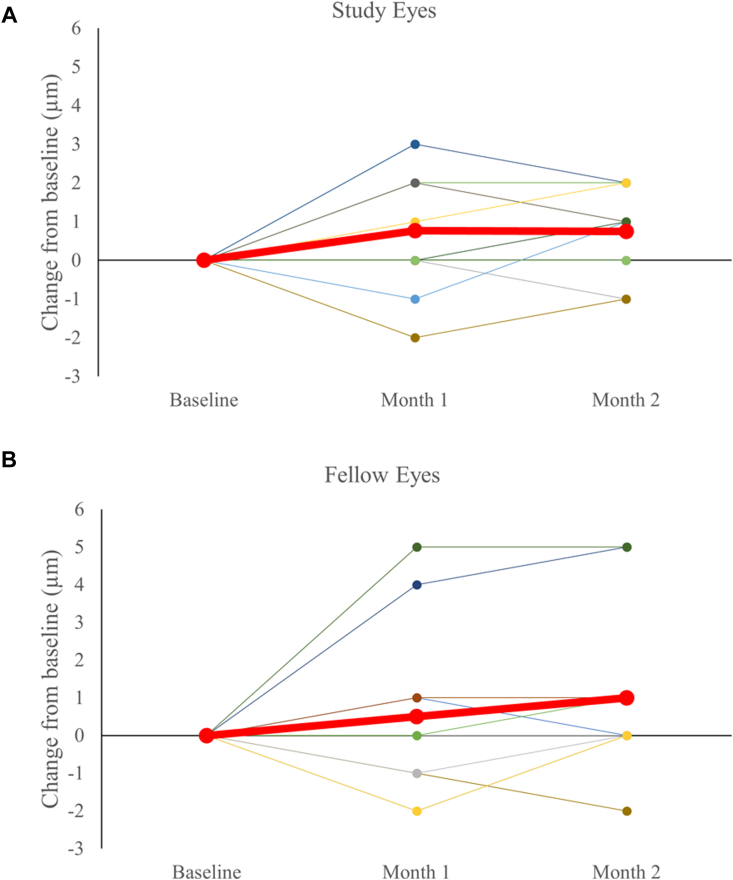
Ganglion cell complex thickness change for group 3 from baseline through month 2 for study (panel **A**, N = 13) and fellow eyes (panel **B**, N = 10). Red line shows mean at each time point.

We detected no significant difference from baseline to month 1 between groups in full thickness macular OCT (study eyes, –1 ± 1.15 μm; fellow eyes 0.6 ± 1.28 μm; Fig 8), or in GCC (study eyes, 0.76 ± 0.42 μm; fellow eyes, 0.50 ± 0.75 μm; Fig 9). The slight increases or decreases in macular thickness did not correlate with changes in RNFL (R^2^ = 0.0001).

### Case Example

**Figure 10:**
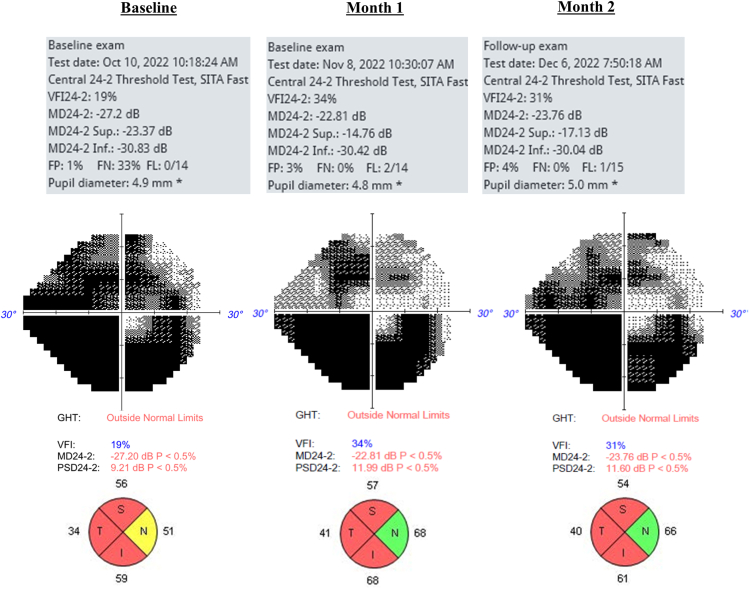
Patient IT-019 visual field and RNFL thickness quadrant data. dB = decibels; FL = fixation losses; FN = false-negative; FP = false-positive; GHT = glaucoma hemifield test; I = inferior quadrant; MD = mean deviation; N = nasal quadrant; PSD = pattern standard deviation; RNFL = retinal nerve fiber layer; S = superior quadrant; T = temporal quadrant; VFI = visual field index.

Patient IT-019 is a 56-year-old White male with a diagnosis of severe chronic angle-closure glaucoma in his right eye, which served as his study eye. He was enrolled and randomly assigned to the 100 u/ml group. The patient reported using the topical insulin reliably for 1 month per protocol, in addition to his topical ocular hypotensive medications, with ocular surface stinging as the only AE reported, which subsided almost immediately after initial insulin distribution. On HVF, an improvement of 4.39 dB in MD was observed (baseline –27.20 dB to month 1 -22.81 dB), which regressed slightly after the 1-month washout period (month 2 -23.76 dB) (Fig 10). A concomitant increase in average RNFL thickness was observed (baseline, 50 μm to month 1, 59 μm), as well as in 3 quadrants, that decreased slightly after washout (month 2, 55 μm) (Fig 10). A structure–function correlation was observed with superior visual field improvement and inferior RNFL thickening during the insulin treatment period. This was the most encouraging case in demonstrating possible structure–function correlation for all patients enrolled in group 3.

## Discussion

In this phase I trial in patients with glaucoma, daily topical insulin at 100 u/ml and 500 u/ml (approximately 4 u/drop and 20 u/drop, based on a 40 μl drop volume) proved to be safe and well tolerated, and a statistically significant increase in RNFL thickness, along with case examples of structure–function–correlated improvement in some patients, together support the plausibility of biological activity at the level of the retina or optic nerve.

Addressing the primary endpoint of safety, there were no serious AEs in patients with glaucoma. The most common adverse effects were temporary and mild in severity, including stinging, watery eye, and dry-eye sensation, all of which subsided immediately after initial application of the drops, and did not persist after cessation of treatment. These effects could be attributable to the insulin itself or to acidity or excipients in the formulation used, as for example meta-cresol has been shown to elicit at least mild ocular symptoms.^15^^,^^16^ No systemic side effects, such as hypoglycemia or hypokalemia, were detected, consistent with prior safety data on topical insulin tested at the lower 100 u/ml concentration.^17^^,^^18^ In studies using diabetic dog and rat models, topical administration of insulin demonstrated a significant decrease in blood D-glucose levels.^19^^,^^20^ However, this effect was not seen in this trial with human participants, as blood glucose levels remained stable throughout the study duration, consistent with previous preclinical studies in mice.^9^^,^^10^ We also did not detect any effects of topical therapy on the fellow eye by any measure, also arguing against systemic distribution. Topical insulin has previously been studied for refractory neurotrophic corneal ulcers, persistent epithelial defects of the cornea, and neurotrophic keratitis at a much lower concentration of 1 u/mL (approximately 0.025 u/drop) dosed every 6 hours from 1 week to 1 month, with no serious AEs, safety concerns, or systemic toxicity reported.^21^, ^22^, ^23^ Our data extend these results on safety and tolerability to the higher doses of 4 u or 20 u per drop.

This study also looked for evidence of biological activity at the retina or optic nerve head. In mice, fluorescently tagged insulin was detected in the retina by immunohistochemistry against the fluorophore as early as 15 minutes after topical administration,^9^ and an approximately sevenfold increase in insulin levels were detected by enzyme-linked immunosorbent assay 30 minutes after insulin eye drop administration.^10^ Together, these findings strongly suggest that insulin reaches the retina, consistent with pharmacokinetic and pharmacodynamics of other topically applied proteins in rodents,^24^ although the much smaller rodent eye with much thinner cornea and sclera may not appropriately mimic drug distribution in the human eye. Recent data from topical application of insulin in a nonhuman primate glaucoma model protected against RNFL thinning in an eye more similar to human and thereby strongly support the plausibility of pharmacodynamic effect.^25^ In our study, the significant thickening in RNFL thickness over 1 month of administration suggests a pharmacodynamic effect of insulin in the retina or optic nerve after topical administration. Furthermore, almost all of the RNFL thickening reverted toward or even all of the way back to baseline after 1 month of washout, with only 3 eyes showing a greater RNFL thickness at month 2 than month 1; it is not clear that this would be attributable to a longer or delayed pharmacodynamic effect. Though less likely, it is plausible that the thickening of the RNFL could be a deleterious effect. Axonal injury due to acute optic neuropathies or early IOP-related injury can cause intracellular cytoskeletal swelling or edema in axons or astrocytes,^26^, ^27^, ^28^ although there was no edema detected on OCT imaging in this study. Thus, these data in human patients strongly argue for pharmacodynamic effect in the posterior segment consistent with the data from topical administration of insulin in animal models.

This effect was specific to the RNFL, as significant thickening of the GCC was not detected, although for this limited study we were not able to segment and analyze the inner plexiform layer/dendrites independent of the RNFL or GCC to further study the RGCs' dendritic layer in the retina. In a preclinical study demonstrating insulin neuroprotection in glaucoma mouse models, therapeutically delivered either by intraperitoneal injection or topically to similar effect, insulin-treated mice demonstrated larger and more complex dendrites.^9^ It will be important to look for evidence of insulin-mediated changes in inner plexiform layer/dendrites, or in metabolic function not seen in 1 month by FPF imaging, in human glaucoma patients in clinical trials incorporating longer treatment duration.

Insulin's potential as a neuroprotective or regenerative therapeutic in glaucoma has gained support from preclinical research in glaucoma^29^ and has also been explored in neurodegenerative diseases such as Alzheimer or Parkinson disease.^8^ Although studied primarily as a hormone that regulates glucose homeostasis, insulin has been shown to affect cognitive function and neuronal survival due to its interaction with insulin-like growth factor receptors, which are expressed by adult RGCs, in addition to canonical insulin receptors.^6^^,^^30^ Insufficient insulin signaling may play a key role in the progression of glaucoma.^31^ Preclinical studies have shown insulin as an important activator of Mechanistic Target of Rapamycin Complexes 1 and 2 (mTORC1 and mTORC2), which broadly promote protein synthesis and cell growth,^32^ regulate RGC growth and regeneration,^9^ and are required for insulin-mediated RGC regeneration and neuroprotection in a mouse glaucoma model.^10^ From our human data, we speculate that the significant increase in RNFL thickness in study eyes is due to action as a growth factor stimulating axon hypertrophy, in addition to changes in other cells such as astrocytes surrounding RGC axons. Similar effects of concomitant neuroprotection and RNFL thickening have also been observed in response to treatment with other neurotrophic factors such as ciliary neurotrophic factor and nerve growth factor, in animal models^33^, ^34^, ^35^ and in patients with human glaucoma.^36^^,^^37^ We hypothesize that the positive effects of insulin in these patients with glaucoma may be mediated by effects on insulin-like growth factor receptors in addition to metabolic effects through glucose regulation, a hypothesis could be tested first in animal models, for example, in mouse models in which insulin-like growth factor receptors have been suppressed or knocked out in RGCs.

In addition to small sample size, there are certain limitations to this study that are of note. In group 3, HVF exams (8 of 102) were missing due to patients failing to complete the test, and OCT scans (9 of 102) were excluded due to low OCT signal strength or segmentation errors. If a patient did not complete reliable scans for either HVF or OCT testing at all study visits, they were excluded from the analysis entirely. However, if a patient completed HVF testing at all visits but not OCT testing (or vice versa), we included their complete HVF data in the analysis while excluding their incomplete OCT data. Additionally, only 1 HVF exam was performed per time point, limiting the amount of data available for analysis and precluding averaging to reduce variability, a preferred approach for clinical trials.^36^^,^^38^^,^^39^ In group 1, topical anesthetic used prior to tonometry may have muted sensation of local reactions such as burning, stinging, or itching. Finally, this was not a true randomized trial with a placebo control, and only the worse eye of each participant received treatment with the contralateral eye serving as control at the group level. When data analysis was limited to only include paired eyes from patients with useable data collected from both eyes, the sample size was too small to reach significance. In a future longer trial, we would suggest randomizing eligible eyes by the unit of the patient and including a placebo comparator.

In conclusion, there is an unmet need for neuroprotective or neuroenhancing treatments for glaucoma. Along with other strategies such as nicotinamide,^40^ recombinant human nerve growth factor,^36^ ciliary neurotrophic factor,^37^ and anticomplement C1q antibody,^39^ topical insulin has a strong scientific premise as a therapeutic for glaucoma. These promising data on the safety and tolerability as well as improvement in RNFL thickness over a 1-month course of treatment, together with strong neuroprotective effects in preclinical studies, encourage the initiation of larger and broader studies to further evaluate topical insulin in glaucoma.
